# Fascin-1 limits myosin activity in microglia to control mechanical characterization of the injured spinal cord

**DOI:** 10.1186/s12974-024-03089-5

**Published:** 2024-04-10

**Authors:** Jinxin Huang, Xuyang Hu, Zeqiang Chen, Fangru Ouyang, Jianjian Li, Yixue Hu, Yuanzhe Zhao, Jingwen Wang, Fei Yao, Juehua Jing, Li Cheng

**Affiliations:** 1 Department of Orthopaedics, The Second Affiliated Hospital of Anhui Medical University, Hefei, 230601 China; 2 Institute of Orthopaedics, Research Center for Translational Medicine, The Second Affiliated Hospital of Anhui Medical University, Hefei, 230601 China

**Keywords:** Spinal cord injury, Microglia, Mechanical characterization, Fascin-1, Myosin

## Abstract

**Background:**

Mechanical softening of the glial scar region regulates axonal regeneration to impede neurological recovery in central nervous system (CNS) injury. Microglia, a crucial cellular component of the glial scar, facilitate neuronal survival and neurological recovery after spinal cord injury (SCI). However, the critical mechanical characterization of injured spinal cord that harmonizes neuroprotective function of microglia remains poorly understood.

**Methods:**

Spinal cord tissue stiffness was assessed using atomic force microscopy (AFM) in a mouse model of crush injury. Pharmacological depletion of microglia using PLX5622 was used to explore the effect of microglia on mechanical characterization. Conditional knockout of Fascin-1 in microglia (*Fascin-1* CKO) alone or in combination with inhibition of myosin activity was performed to delve into relevant mechanisms of microglia regulating mechanical signal. Immunofluorescence staining was performed to evaluate the related protein levels, inflammatory cells, and neuron survival after SCI. The Basso mouse scale score was calculated to assess functional recovery.

**Results:**

Spinal cord tissue significantly softens after SCI. Microglia depletion or Fascin-1 knockout in microglia limits tissue softening and alters mechanical characterization, which leads to increased tissue pathology and impaired functional recovery. Mechanistically, Fascin-1 inhibits myosin activation to promote microglial migration and control mechanical characterization after SCI.

**Conclusions:**

We reveal that Fascin-1 limits myosin activity to regulate mechanical characterization after SCI, and this mechanical signal should be considered in future approaches for the treatment of CNS diseases.

**Supplementary Information:**

The online version contains supplementary material available at 10.1186/s12974-024-03089-5.

## Background

Spinal cord injury (SCI) is a devastating central nervous system trauma that leads to severe sensory and motor dysfunction, reducing the quality and longevity of life for patients [1, 2]. Astrocytes, a type of macroglial cells that provide support, nutrition, and protection for neurons, migrate to the injury lesion and form a physical barrier of glial scar, which limits the spread of inflammatory cells and promotes axonal regeneration during the chronic phase after SCI [3, 4]. The inflammatory response gives rise to secondary injury, establishing a prolonged and chronically inflamed environment at the injury site, which can hinder axonal regeneration and promote glial scar formation. For decades, the studies on the central nervous system (CNS) axonal regeneration have focused on chemical signals such as biochemistry, molecular biology, and genetics [5, 6]. It is increasingly recognized that many biological systems also integrate mechanical factors [7–9]. Notably, astrocyte scar is softer than uninjured tissue regions in acute and subacute phase [10]. The specific composition of extracellular matrix (ECM) in glial scar containing collagen I and proteoglycans, glial intermediate filaments containing glial fibrillary acidic protein (GFAP) and vimentin, as well as the cellular composition such as activated astrocytes were considered to the potential factor of scar softening [11]. The mechanical properties are essential determinant of neuronal development, axon regeneration, and tissue repair [12]. A detailed characterization of the mechanical signal characterization at the injury site is pivotal for understanding the immense complexity of the immune environment in SCI, and for developing therapeutic strategies that preferentially alters the mechanical signal to promote axonal regeneration and functional recovery.

Microglia, as a unique and main inflammatory cell at the site of injury, play a pivotal role in various processes, including myelin phagocytosis, neuronal survival, inflammatory responses, and glial scar formation [13–15]. Pharmacologically depleted microglia using PLX5622, a colony-stimulating factor 1 receptor (CSF1R) inhibitor, has been shown to disrupt glial scar formation and inflammation cell diffusion, which is detrimental to neuronal survival [13]. Furthermore, single-cell RNA sequencing (scRNA-seq) results elucidate the critical significance of microglia in coordinating the damage response of resident glial cells and infiltrating mononuclear cells after SCI. However, it remains unknown whether microglia are involved in the alteration of mechanical signals at the injury site.

Fascin-1 is a member of the Fascin family gene encoding actin-bundling protein, which stabilizes filopodia and invader feet by regulating the parallel bundling of actin filaments and regulates cell migration, invasion, and adhesion [16, 17]. Our previous study showed that Fascin-1 exhibits a significant and specific expression in microglia after SCI, and confirmed that Fascin-1 induces the migration of microglia in vitro [18]. Recent research has suggested that migrating border cells could regulate the mechanical characterization of corresponding microenvironment in *Drosophila*. Mechanistically, the actin-bundling protein Fascin-1 limits myosin activity to promote migration and control substrate stiffness in *Drosophila* border cells [19]. We thus speculate that upregulated Fascin-1 may suppress the level of active myosin in microglia, decreasing the stiffness of the injured spinal cord.

In this study, we detected the mechanical properties of the penumbra region and injured lesion core of spinal cord tissue and found that spinal cord tissue would soften after crush injury. Surprisingly, there was no difference in mechanical signals between the penumbra and the injured lesion core of spinal cord tissue. Microglia elimination or genetic Fasin-1 deficiency in microglia limits tissue softening characterized by enhancing elastic properties, which resulted in reduced neuronal survival and impaired functional recovery. In genetic Fasin-1 deficiency in microglia mice, pharmacological inhibition of myosin activity induces a re-softening of the injured spinal cord, promoting neuronal survival and functional recovery. This reveals a mechanism wherein Fascin-1 regulates myosin activity to facilitate microglial migration. Thus, our study has revealed the role of the Fascin-1/Myosin pathway in mediating microglial migration in mammalian SCI, leading to tissue softening and promoting neuronal survival and functional recovery.

## Methods

### Animals

The C57BL/6J mice of the wild-type were acquired from the Experimental Animal Centre of Anhui Medical University. The C57BL/6J background *CX3CR1*^*cre*^ mice (NM-KI-200,079) and *Fascin-1*^*fl/fl*^ mice (NM-CKO-210,023) were obtained from Shanghai Model Organisms Center and crossed to generate *CX3CR1*^*cre*^; *Fascin-1*^*fl/fl*^ (*Fascin-1* CKO) mice. Experiments were performed on mixed gender adult mice (20 ± 2 g) at 8–10 weeks. Animal experiments conducted at Anhui Medical University adhered to the applicable guidelines and were approved by the Animal Ethics Committee (Approval No. LLSC20220381). Mice were randomly assigned and housed in pathogen-free microbial cages with enough food and water, following a 12-h light/dark cycle.

### SCI model establishment

A spinal cord crush injury model was established based on our previous method [20]. In short, the mice were anaesthetized using sodium pentobarbital (50 mg/kg). The spinal cord was dissected to expose at the T10 level and compressed continuously for 5 s with sterile Dumont #5 forceps (11252-20, Fine Science Tools, Germany). Mice were treated with analgesia and antibiotics after surgery. The bladder was manually emptied twice daily postoperatively to prevent infection.

### Microglia elimination

To eliminate microglia, mice were intraperitoneally injected twice daily with the CSF1R inhibitor PLX5622 (50 mg/kg, MedChemExpress, HY-114,153) starting three days before surgery until 14 days post-injury (dpi). PLX5622 was dissolved in a solution of 5% DMSO, 30% polyethylene glycol 300, 10% polysorbate 80, and 55% saline, according to the manufacturer’s instructions. An equal volume of drug solvent was used as a control.

### Intraperitoneal injection of Y-27,632

Mice were administered Y-27,632 (10 mg/kg, MedChemExpress, HY-10,071) through intraperitoneal injection on a daily basis from 4 h post-injury until the time of sacrifice. Y-27,632 was diluted in a solution of 10% DMSO, 40% polyethylene glycol 300, 5% polysorbate 80, and 45% saline, according to the manufacturer’s instructions. An equal volume of drug solvent was used as the control.

### Dissection and preparation of tissue

Sodium pentobarbital (50 mg/kg) was used to anesthetize the mice. Fresh spinal cord tissue was removed after perfusion with cold PBS solution and snap frozen. Using a cryostat (NX50, Thermo Fisher Scientific, USA), spinal cord tissue was sliced into sagittal sections that were 20 μm-thick and continuous. Freshly frozen tissue sections were affixed onto slides and stored at -80℃. The frozen/thawed sample preparation process did not affect the mechanical properties of the spinal cord [21]. The spinal cord slices used for atomic force microscopy (AFM) were pretreated for immunofluorescence staining.

### Atomic force microscopy

The slides with tissue sections were gradually equilibrated to room temperature for 15–30 min in a petri dish before commencing the AFM measurements, ensuring constant high humidity to prevent desiccation. To evaluate the mechanical properties of the spinal cord by AFM, we selected areas of interest by lesion core extent using an atomic force microscope (Bruker Dimension ICON). A tipless silicon cantilever (Bruker, spring constant = 0.24 N m^− 1^) was glued with polystyrene beads (diameter = 22 μm) to the tip of the cantilever. AFM microindentation force profiles were conducted at room temperature in PBS for each region of interest. Force-distance curves were taken with the set force of 5 nN and speed of 2 μm s^− 1^. A grid heatmap was subsequently generated to depict the modulus of elasticity, which extracted from force-distance curves by the Hertz model [22]. The small deviation and reliability of the Hertz model were calculated and assessed with MATLAB [23].

### Immunofluorescence staining

A mixture of 0.3% Triton X-100 (Solarbio, T8200) and 10% donkey serum albumin (DSA, Solarbio, SL050) was applied to block the sections for 60 min at room temperature. The primary antibodies incubated with the spinal cord sections at a temperature of 4℃ for the entire night. We utilized the following primary antibodies: mouse anti-Fascin-1 (1:50, Santa Cruz, sc-21,743), rabbit anti-CX3CR1 (1:100, Novus, NBP1-76872), rabbit anti-NeuN (1:500, Abcam, ab177487), rat anti-GFAP (1:200, Invitrogen, 13–0300), rabbit anti-Vimentin (1:200, Abcam, ab92547), rabbit anti-Connexin43 (Cx43, 1: 200, Proteintech, 26980-1-AP), rat anti-CD68 (1:400, Bio-Rad, MCA1957), and mouse anti-phospho-Myosin Regulatory Light Chain 2 (Ser19) (pMRLC, 1:200, Cell signaling, #3671). Secondary antibodies were diluted with PBS containing 1% DSA and incubated with sections for 60 min at room temperature. Alexa Fluor 555 and Alexa Fluor 488 (1:500, Invitrogen, A-31,572, A-21,202, and A-21,208) were used as the secondary antibodies. Finally, DAPI (Beyotime Biotechnology, C1005) were used to label the nuclei.

In addition, the cells were fixed by treating with 4% PFA for 10 min, 0.5% Triton X-100 in PBS was used to permeabilize for 10 min. Subsequently, they were blocked with PBS containing 5% DSA for 30 min at room temperature. The above-mentioned primary antibodies were diluted in 1% DSA in PBS and incubate with cells at a temperature of 4℃ for the entire night. The above-mentioned secondary antibodies were diluted with PBS containing 1% DSA and left to incubate for 60 min at room temperature. For each sagittal section, 6 areas of view were randomly selected for statistics analysis. The sections were imaged using an Axio Scope A1 fluorescence microscope system(Zeiss, Germany) with ZEN 3.3 software.

### Astrocyte culture

Based on previous instruction [24] and our measured elastic modulus, hydrogel substrates with elastic moduli at 200 Pa and 700 Pa were first obtained by mixing 40% acrylamide and 3% bisacrylamide in different proportions and tested by AFM in agreement with the previous method [25]. The astrocyte cell line was acquired from the American Collection of Exemplary Cultures (CRL-2006). Astrocytes were cultured in DMEM (Servicebio, G4511), a modified version of Dulbecco’s Eagle medium, enriched with 10% fetal bovine serum (FBS, Gibco, 10,270,106) and supplemented with 1% penicillin-streptomycin (Beyotime, C0222). Astrocytes were placed in an incubator with a temperature of 37℃, which had a mixture of 95% O2 and 5% CO2, then inoculated on hydrogel substrates of different stiffnesses.

### Primary microglia culture

Primary microglia were obtained using the protocol previously published [26]. Briefly, cerebral cortices from 8 weeks mice brains were dissected without dura. The remaining brain tissues were dissociated by enzymatic digestion with a Neural Tissue Dissociation Kit (P) (Miltenyi Biotec). The mixed cells were resuspended in DMEM with the addition of 10% FBS and filtrated using a 70-µm cell strainer (Falcon). After being cultured for 2 weeks, primary microglia were collected by shaking for 30 min at a speed of 200 rpm. Within 24 h of collection, the microglia were exposed to different treatments.

### Transwell assay

Primary microglia were subjected to invasive migration assay by Transwell chamber (Costar, 3415). Cell suspensions without serum mixed with Y-27,632 (5 µM, MedChemExpress, HY-10,071) and inoculated in the upper chamber at a concentration of 1 × 10^5^ cells per chamber. The control serum-free medium was mixed with an equal amount of 0.1% DMSO. The lower chambers were supplemented with medium containing 10% FBS. After 24 h, microglia suspended in the small chamber were removed with cotton swabs and then fixed with 4% formaldehyde for 20 min. Finally, microglia were dyed using 0.1% crystal violet for a duration of 15 min and then rinsed thrice with PBS.

### Scratch assay

Primary microglia were inoculated in 6-well plates until the density reached 90–100%. Serum-free mediums containing 5 µM Y-27,632 were added to the experimental group, and serum-free mediums containing PBS were used as a control treatment. Vertical scratches were made on the cell monolayers with a sterile gun tip, and the scratched cells were washed off with PBS after discarding the medium. The extent of microglial migration at 0 h and 24 h was photographed under an orthotopic microscope.

### Behavioral assessment

All mice were trained for 3 days to test their adaptation before behavioral analysis. The testing was conducted in a blind manner by two experienced researchers. Using an open-field rating scale, Basso mouse scale (BMS) score evaluations were conducted before the injury and at 1, 3, 7, and 14 dpi, following the protocol established by Basso and his team [27]. Scores ranged from 0 to 9, and the average of the two researchers’ scores determined the final score for each mouse, with 8 animals per group.

### Image acquisition and quantitative analysis

To determine the elastic modulus after SCI, Two points in the GFAP^−^ lesion core and GFAP^+^ penumbra region (approximately 150–300 μm away from the injury) were measured in 3 animals per time point.

To determine the treatment effect of targeting microglia, the result of each sample take the average of 3 sections, and the count was obtained by analyzing 3 animals per group. To assess the effect of PLX5622 in clearing microglia, the number of CX3CR1^+^ cells per mm^2^ was counted using a 10-fold objective lens. To determine the effect of conditional deletion of Fascin-1 in microglia, the Fascin-1^+^CX3CR1^+^ cells were counted and expressed as the number of Fascin-1^+^CX3CR1^+^ cells per mm^2^. To quantify microglial migration in the spinal cord, the CX3CR1^+^ cells per mm^2^ was counted at various distances from the lesion epicenter at 7 and 14 dpi.

To quantify the expression of different proteins in the spinal cord, 5 sections in each sample were stained for GFAP, vimentin, and Cx43, with 3 animals per group. The GFAP^+^, vimentin^+^, and Cx43^+^ areas were measured by ImageJ and expressed as the proportional area occupied by staining in tissue sections. For the expression of GFAP, vimentin, and Cx43 in astrocytes in vitro under different mechanical stresses, the 6 experimental data per group from astrocytes cultured on 200 Pa and 700 Pa was analyzed on 40×images and expressed as relative fluorescence intensity between two groups.

To assess the pMRLC levels in microglia, 3 sections in each sample were stained for pMRLC and CX3CR1, with 3 animals per group. The pMRLC^+^CX3CR1^+^ cells and CX3CR1^+^ cells was counted using ImageJ in selected microglial scar region, and the data are expressed as a percentage of pMRLC^+^CX3CR1^+^ cells to the overall CX3CR1^+^ cells.

To assess tissue pathology in the injured spinal cord, 5 sections in each sample were stained for CD68, with 3 animals per group. The different sizes of CD68^+^ cells were tallied and presented as the percentage of different sizes of CD68^+^ cells to total CD68^+^ cells. To evaluate the neuronal survival, as previously described [20], the sections were categorized into three regions, namely Z1 (0–250 μm), Z2 (250–500 μm), and Z3 (1000–1250 μm), depending on their distance from the lesion core. The NeuN^+^ cells was counted by staining the sagittal sections with GFAP and NeuN, and expressed as the number of NeuN^+^ cells per mm^2^.

### Statistical analysis

Sample sizes were determined based on prior experience, without the utilization of statistical methods to predetermine them. The data is presented as mean ± standard error of the mean (SEM). Multiple comparisons were assessed using either one-way or two-way analysis of variance (ANOVA) followed by Tukey’s post hoc test or Bonferroni post hoc correction. The two-tailed Student’s t-test was employed to compare two groups. GraphPad Prism 9.0 was used for data analysis and graphing, and statistical significance was determined with a significance threshold of *p* < 0.05.

## Results

### Spinal cord tissue softens after crush injury

**Fig. 1:**
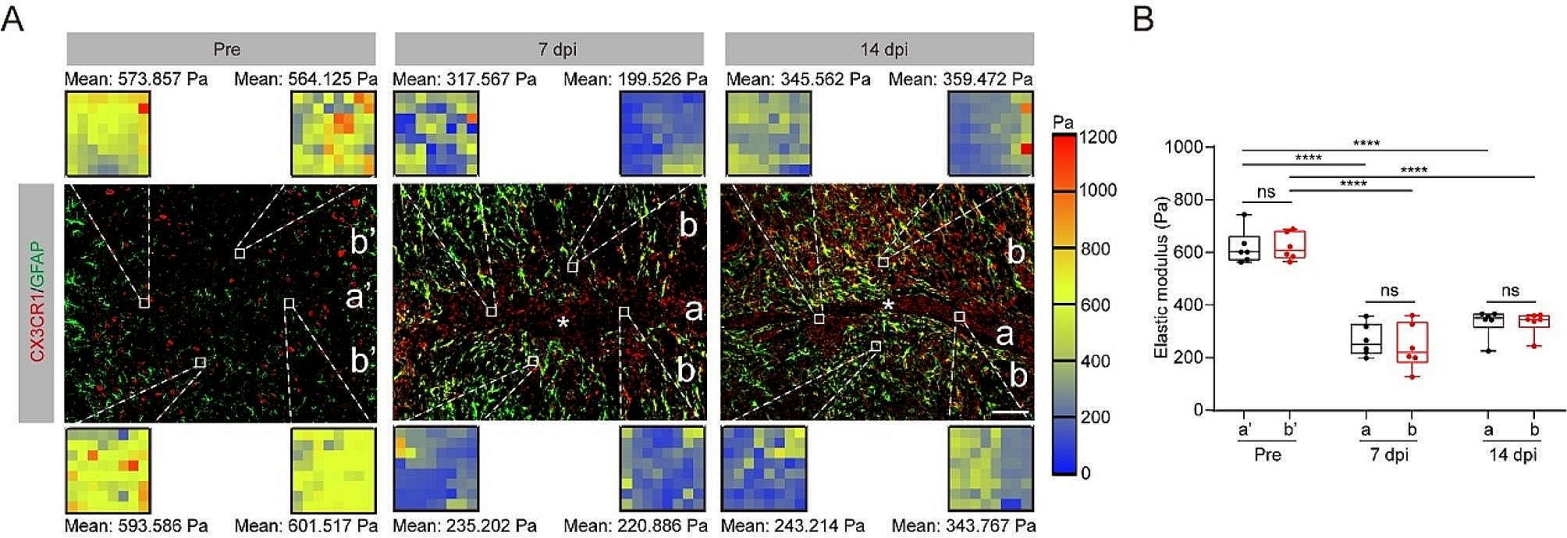
Spinal cord tissue softens after crush injury. (**A**) Sagittal immunofluorescence images for microglia (CX3CR1, red) and astrocyte (GFAP, green) in spinal cord sagittal sections from uninjured mice and injured mice at 7 and 14 days post-injury (dpi). The color maps represent the elastic modulus in the regions of interest (ROI). The lesion core was marked with the asterisks. Point a represents the GFAP^−^ lesion core, and point b represents the GFAP^+^ penumbra region from injured mice at 7 and 14 dpi. The positions of uninjured mice a’ and b’ were similar to those of a and b at 7 and 14 dpi, respectively. Scale bar: 100 μm. (**B**) Comparison of the elastic properties of lesion core and penumbra area in (**A**). *n* = 3 animals in (**B**). ns, no significance; ****P < 0.0001 by two-way ANOVA

Microglia are swiftly recruited around the lesion core within two weeks after SCI, forming a dense “microglial scar” at the astrocyte-immune cell interface [13]. To investigate the correlation between microglial migration and spatiotemporal changes in mechanical properties of the spinal cord following a crush injury, we first performed AFM microindentation and immunofluorescence staining for the injured spinal cord at 7 and 14 dpi. Immunostaining for CX3CR1 label microglia in the spinal cord [28, 29]. In normal mouse spinal cord, CX3CR1^+^ microglia and GFAP^+^ astrocytes are widely distributed in parenchyma. GFAP^+^ astrocytes form glial scar in the penumbra region, while CX3CR1^+^ microglia migrate and distribute within the lesion core and penumbra after SCI as shown in Fig. 1A. Due to this characteristic distribution of microglia and astrocytes, we delineate two tissue areas: regions a and b, corresponding to the GFAP^−^ lesion core and the GFAP^+^ penumbra region from injured mice. The detection sites of the uninjured tissues, designated as a’ and b’, exhibit similarities with the regions of the damaged tissues in relation to the spinal cord midline. Each area generated two representative stiffness maps. Elastic modulus values, which assess the mechanical characteristics of the tissue, ranged from 550 to 750 Pa in uninjured spinal cord tissue. However, the elastic modulus in the lesion core (region a) and penumbra (region b) decreased by 57% and 61% at 7 dpi, respectively, compared to regions a’ and b’ in the uninjured spinal cord tissue (Fig. 1A, B). Similarly, we noticed a significant decrease in the elastic modulus of both the lesion core and the penumbra compared to regions a’ and b’ in uninjured spinal cord tissue at 14 dpi, which is similar to the results of a previously published rat spinal cord crush injury model [30]. Surprisingly, no notable mechanical distinction was observed between the lesion core and penumbra region at 7 and 14 dpi (Fig. 1B). Thus, these results suggest that spinal cord tissues undergo softening following spinal cord crush injury, and microglial migration may be involved in the glial scar-mediated spinal cord softening after SCI.

### Microglia depletion limits tissue softening after SCI

**Fig. 2:**
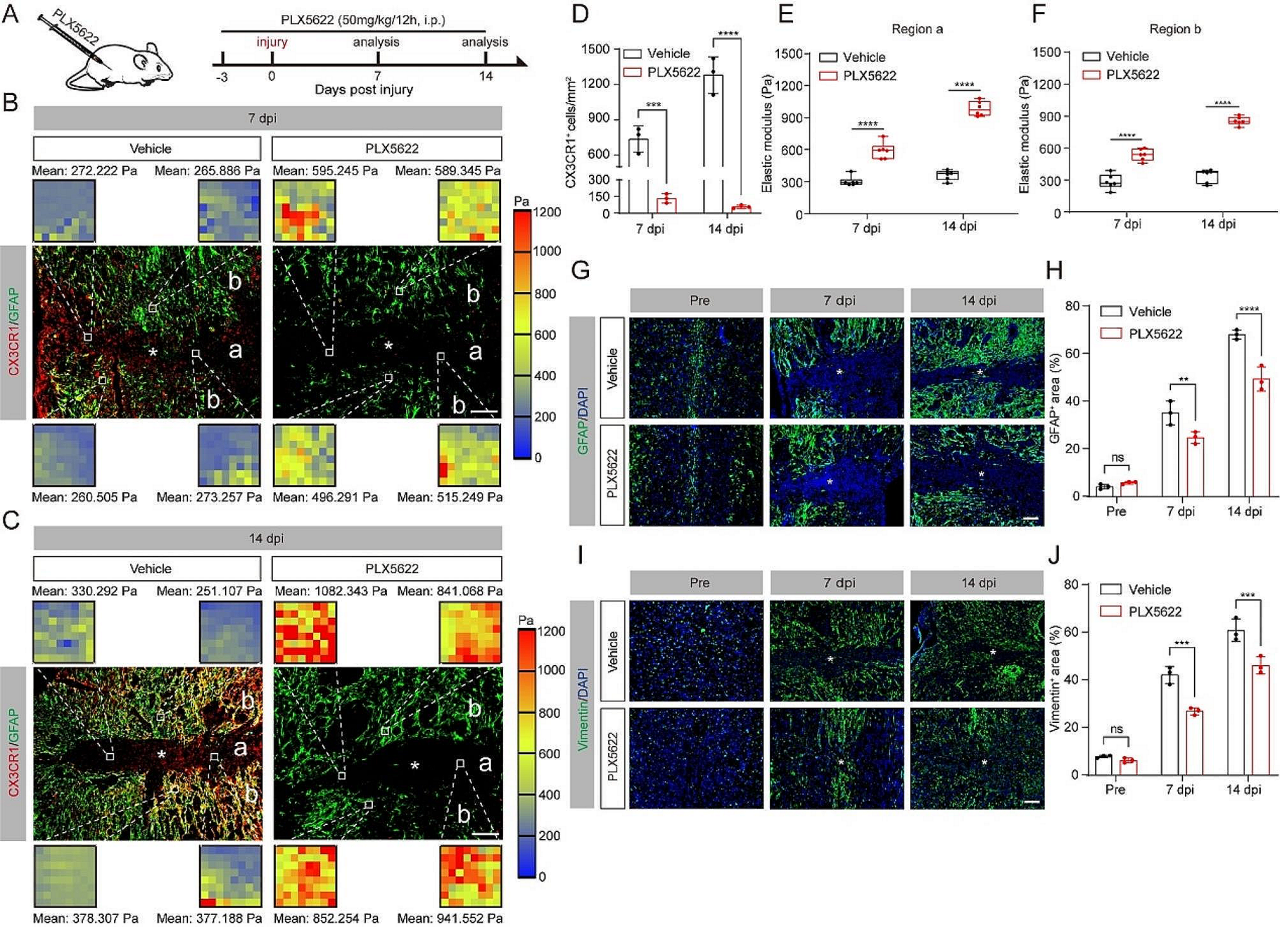
Depletion of microglia limits tissue softening after SCI. (**A**) Schematic of the mice treated with PLX5622. The CSF1R inhibitor PLX5622 was administered by intraperitoneal injection twice daily from 3 days before injury to 14 dpi. (**B**, **C**) Sagittal immunofluorescence images for microglia (CX3CR1, red) and astrocyte (GFAP, green) from injured mice treated with vehicle and PLX5622 at 7 (**B**) and 14 dpi (**C**). Region a represents the GFAP^−^ lesion core, and region b represents the GFAP^+^ penumbra region. Scale bar: 100 μm. (**D**) Quantitative analysis of the density of microglia in vehicle and PLX5622 groups. (**E**, **F**) Comparison of the elastic properties of lesion core (a, **E**) and penumbra region (b, **F**) in mice treated as described above shown in (**B**) and (**C**). (**G**, **I**) Sagittal immunofluorescence images for the astrocyte markers GFAP (green, **G**) and vimentin (green, **I**) from uninjured mice and injured mice treated with vehicle and PLX5622 at 7 and 14 dpi. The nuclei were marked with DAPI (blue). Scale bar: 100 μm. (**H**, **J**) Percentage quantification of GFAP^+^ area (**H**) and vimentin^+^ area (**J**). The lesion core was marked with the asterisks. *n* = 3 animals in (**D**), (**E**), (**F**), (**H**), and (**J**). ns, no significance; **P < 0.01, ***P < 0.001, ****P < 0.0001 by two-way ANOVA

To investigate the role of microglial migration on tissue softening after SCI, mice were treated with PLX5622 to clear microglia starting from 3 days before SCI and lasting until sacrifice at 14 dpi (Fig. 2A). The immunofluorescence staining results for microglia suggest the efficiency of PLX5622 in clearing microglia reached 82% and 95% at 7 and 14 dpi, respectively (Fig. 2B-D). We found that the elastic modulus of lesion core (region a) and penumbra (region b) increased by 1-1.5 fold in mice that received PLX5622 (Fig. 2B, C, E, F), indicating microglia play a crucial role in the tissue softening process after SCI.

Given that tissue softening has a positive correlation with the upregulation of GFAP and vimentin [11], we next investigated these protein expression levels in mice that received PLX5622 after SCI. In accordance, we observed a significant reduction of GFAP^+^ and vimentin^+^ areas in mice that received PLX5622 at 7 and 14 dpi after SCI (Fig. 2G-J). This directly confirmed that the elimination of microglia disrupts the formation of glial scar. Overall, these results confirm the involvement of microglial migration in the process of softening observed in the lesion core and penumbra after SCI.

### Limited tissue softening by microglia depletion upregulates Cx43 and hinders functional recovery after SCI

**Fig. 3:**
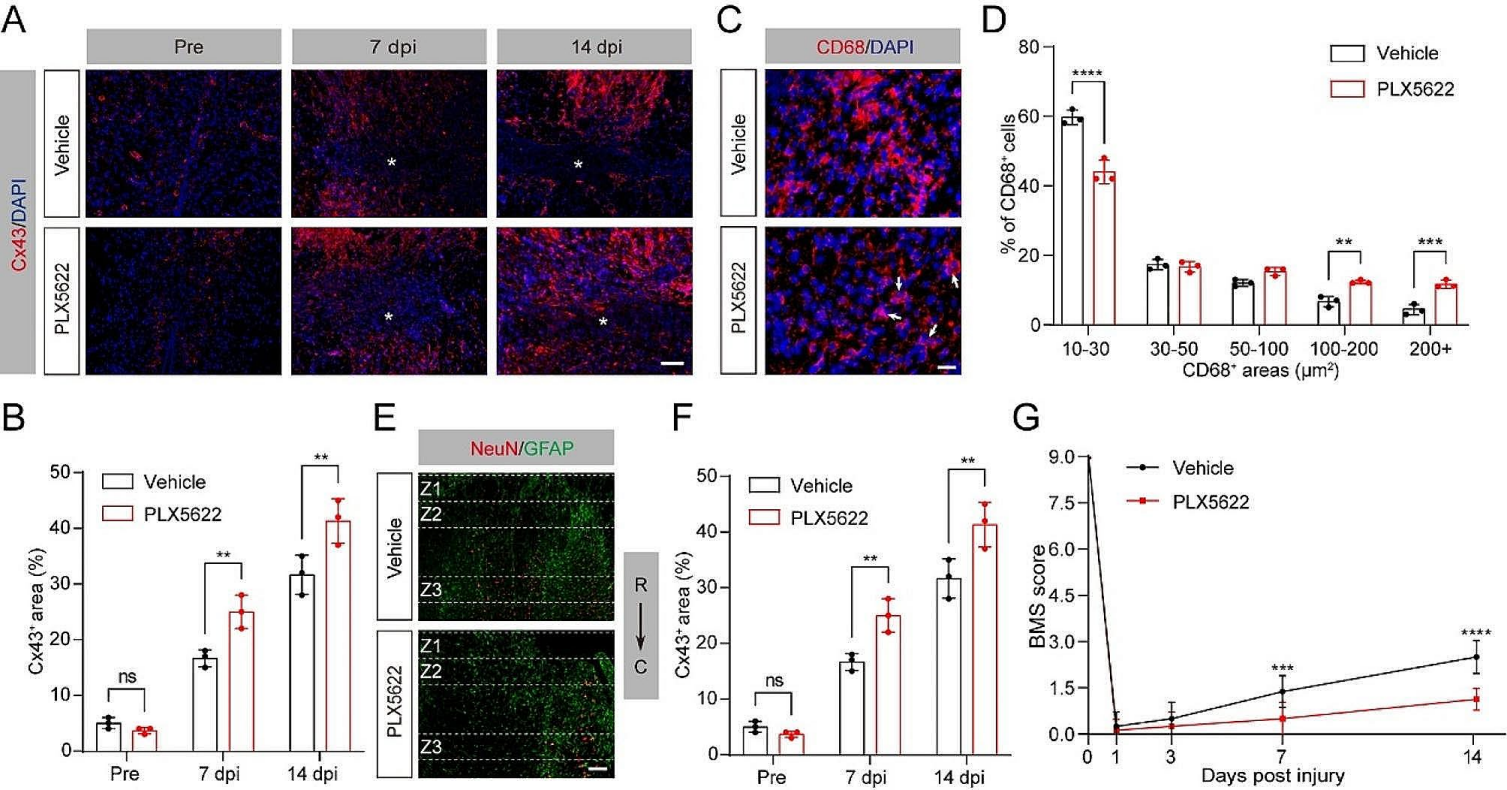
Microglia depletion alters mechanical characterization after SCI. (**A**) Sagittal immunofluorescence images for the Cx43 (red) and DAPI (blue) from uninjured mice and injured mice treated with vehicle and PLX5622 at 7 and14 dpi. The lesion core was marked with the asterisks. Scale bar: 100 μm. (**B**) Percentage quantification of Cx43^+^ area. (**C**) Typical immunofluorescence images of CD68 (red) and DAPI (blue) in the penumbra region from injured mice treated as above at 14 dpi. Scale bar: 20 μm. (**D**) Percentage quantification of CD68^+^ area in different sizes. (**E**) Sagittal immunofluorescence images of NeuN^+^ neurons (red) and GFAP^+^ astrocytes in Z1-Z3 zones neighboring the central lesion core in mice treated as above at 14 dpi. Scale bar: 200 μm. (**F**) Quantification the numbers of NeuN^+^ neurons. (**G**) The BMS score was used to evaluate locomotor function at 0, 1, 3, 7, and 14 dpi between vehicle and PLX5622 groups. *n* = 3 animals in (**B**), (**D**), and (**F**). *n* = 8 animals in (**G**). ns, no significance; *P < 0.05, **P < 0.01, ***P < 0.001, ****P < 0.0001 by two-way ANOVA

Cx43 forms astrocytic gap junctions and hemichannels that can respond to mechanical loading and participate in mechanotransduction [31]. We next assessed the Cx43 expression in mice that received PLX5622 and found that the Cx43 levels were dramatically higher in mice that received PLX5622 than the mice treated with vehicle at 7 and 14 dpi (Fig. 3A, B), indicating the limited tissue softening by microglia depletion upregulate Cx43 after SCI. Induced Cx43 causes excessive inflammation, damaged neurons, and impaired functional recovery [32–34]. Subsequently, we assessed whether the inhibition of spinal cord softening by microglia depletion regulates inflammation, neuron survival, and functional recovery. Immunofluorescent staining results showed that the size of CD68^+^ phagolysosomes was larger in mice that received PLX5622 than the mice treated with vehicle and reminiscent of the ‘foamy’ macrophages connected to neurotoxicity [29, 35] (Fig. 3C, D). As previously described, we utilized the neuronal marker NeuN to label viable neurons in specific regions (Z1-Z3) neighboring to the lesion core of the spinal cord [20]. In mice treated with PLX5622, there was a a significant decrease in the count of viable NeuN^+^ neurons in the Z1-Z3 zones than mice treated with vehicle (Fig. 3E, F). Furthermore, the mice receiving PLX5622 treatment showed significantly worse recovery in locomotor function compared to the vehicle group mice, as assessed by behavioral analysis using the BMS score (Fig. 3G). These results demonstrate that limited tissue softening by microglia depletion upregulates Cx43, exacerbates spinal cord damage pathology, and hinders functional recovery after SCI.

### Fascin-1 deletion in microglia limits tissue softening after SCI

**Fig. 4:**
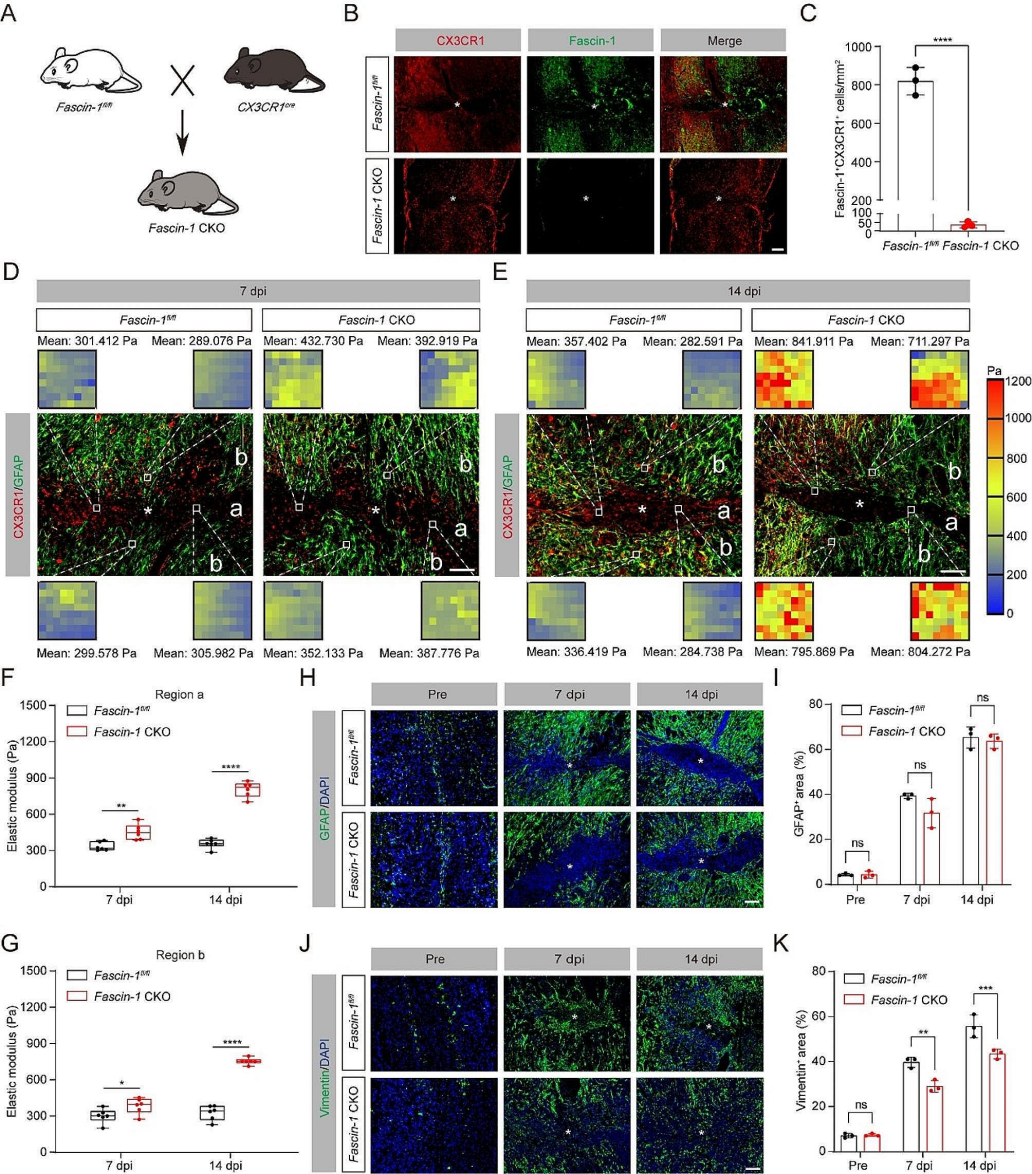
Conditioned knockout of microglial Fascin-1 restricts tissue softening after SCI. (**A**) Construction diagram of *Fascin-1* CKO mice. (**B**) Sagittal immunofluorescence images of CX3CR1 (red) and Fascin-1 (green) from *Fascin-1*^*fl/fl*^ and *Fascin-1* CKO mice at 14 dpi. Scale bar: 200 μm. (**C**) Quantitative analysis of the density of Fascin-1^+^CX3CR1^+^ microglia. (**D, E**) Sagittal immunofluorescence images for microglia (CX3CR1, red) and astrocyte (GFAP, green) from *Fascin-1*^*fl/fl*^ and *Fascin-1* CKO mice at 7 (**D**) and 14 dpi (**E**). Region a represents the GFAP^−^ lesion core, and region b represents the GFAP^+^ penumbra region. Scale bar: 100 μm. (**F**, **G**) Comparison of the elastic properties of lesion core (a, **F**) and penumbra region (b, **G**) in two groups of mice as described above in (**D**) and (**E**). (**H**, **J**) Sagittal immunofluorescence images for the astrocyte marker GFAP (green, **H**) and vimentin (green, **J**) in *Fascin-1*^*fl/fl*^ and *Fascin-1* CKO groups before SCI and at 7 and14 dpi. The nuclei were marked with DAPI (blue). Scale bar: 100 μm. (**I**, **K**) Percentage quantification of GFAP^+^ area (**I**) and vimentin^+^ area (**K**). The lesion core was marked with the asterisks. *n* = 3 animals in (**C**), (**F**), (**G**), (**I**), and (**K**). ns, no signifcance; ****P < 0.0001 by Student’s *t* test in (**C**). *P < 0.05, **P < 0.01, ***P < 0.001, ****P < 0.0001 by two-way ANOVA in (**F**), (**G**), (**I**), and (**K**)

Our previous study showed that Fascin-1 exhibits a significant and specific expression in microglia after SCI, and promotes the migration of microglia in vitro [18]. Recent research has unveiled that Fascin-1 promotes migration of border cells and controls substrate stiffness in *Drosophila* [19]. We thus speculate that Fascin-1 may mediate the mechanical signal changes induced by microglia depletion mentioned above. To confirm the role of Fascin-1 in regulating microglial migration in the context of tissue softening, a conditional Fascin-1 knockout in the microglia (*Fascin-1* CKO) mice was generated (Fig. 4A). It is important to note that the construction of *Fascin-1* CKO mice with gene ablation may also induce deletion of Fascin-1 in CX3CR1-expressing perivascular and blood-derived macrophages [36, 37]. Given that Fascin-1 exhibits a significant and specific expression in microglia after SCI [18], we are confident that our *Fascin-1* CKO mice are credible tools for microglia-specific Fascin-1 ablation. We confirmed that Fascin-1 was successfully knocked out in microglia of the spinal cord (Fig. 4B, C). Compared with *Fascin-1*^*fl/fl*^ mice, the elastic modulus of lesion core (region a) and penumbra (region b) in *Fascin-1* CKO mice was significantly increased at both 7 and 14 dpi (Fig. 4D-G). We further examined the expression of GFAP and vimentin using immunofluorescence staining, and the results showed no obvious distinction in GFAP levels between *Fascin-1*^*fl/fl*^ and *Fascin-1* CKO mice (Fig. 4H, I), which may be explained by the intricate microenvironment after SCI. In contrast, the vimentin^+^ areas in *Fascin-1* CKO mice were significantly lower than in *Fascin-1*^*fl/fl*^ mice at 7 and 14 dpi (Fig. 4J, K). These results indicate that the regulation of microglia migration by Fascin-1 is essential for tissue softening after SCI.

### Limited tissue softening by Fascin-1 deletion in microglia upregulates Cx43 and hinders functional recovery after SCI

**Fig. 5:**
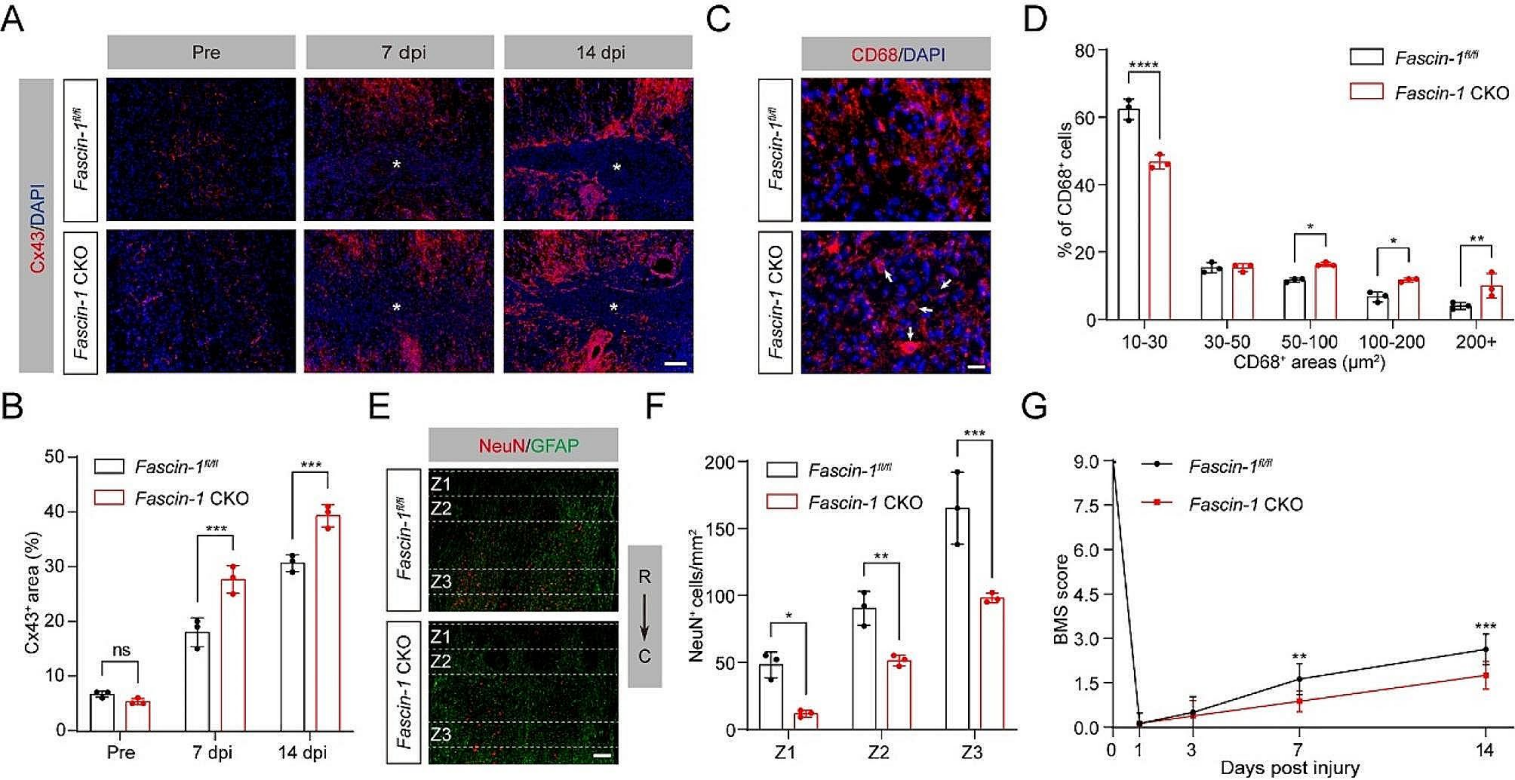
Fascin-1 deletion in microglia alters mechanical characterization after SCI. (**A**) Sagittal immunofluorescence images for the Cx43 (red) and DAPI (blue) from *Fascin-1*^*fl/fl*^ and *Fascin-1* CKO mice before SCI and at 7 and14 dpi. The lesion core was marked with the asterisks. Scale bar: 100 μm. (**B**) Percentage quantification of Cx43^+^ area. (**C**) Typical immunofluorescence images of CD68 (red) and DAPI (blue) in the penumbra region from two groups of mice as described above at 14 dpi. Scale bar: 20 μm. (**D**) Percentage quantification of CD68^+^ area in different sizes. (**E**) Sagittal immunofluorescence images of NeuN^+^ neurons (red) and GFAP^+^ astrocytes (green) in Z1-Z3 zones neighboring the central lesion core in two groups at 14 dpi. Scale bar: 200 μm. (**F**) Quantitative analysis of NeuN^+^ neurons. (**G**) The BMS score was used to evaluate locomotor function at 0, 1, 3, 7, and 14 dpi between *Fascin-1*^*fl/fl*^ and *Fascin-1* CKO groups. *n* = 3 animals in (**B**), (**D**), and (**F**). *n* = 8 animals in (**G**). ns, no significance; *P < 0.05, **P < 0.01, ***P < 0.001, ****P < 0.0001 by two-way ANOVA

Having shown that Fascin-1 deletion in microglia limits tissue softening after SCI, we next investigated whether Fascin-1 in microglia regulates Cx43 expression, tissue pathology, and functional recovery. Similar to the results described above (shown in Fig. 3), the level of Cx43 in *Fascin-1* CKO mice was dramatically upregulated compared to *Fascin-1*^*fl/fl*^ mice (Fig. 5A, B). Accordingly, the conditional deletion of Fascin-1 in microglia induced more larger-sized CD68^+^ phagolysosomes, less neuron survival, and impaired functional recovery (Fig. 5C-G). These data demonstrate that suppressed tissue softening by conditional Fascin-1 deletion in microglia upregulates Cx43 and hinders functional recovery after SCI.

### Physiologic changes in substrate stiffness alter Cx43 expression in astrocytes in vitro

**Fig. 6:**
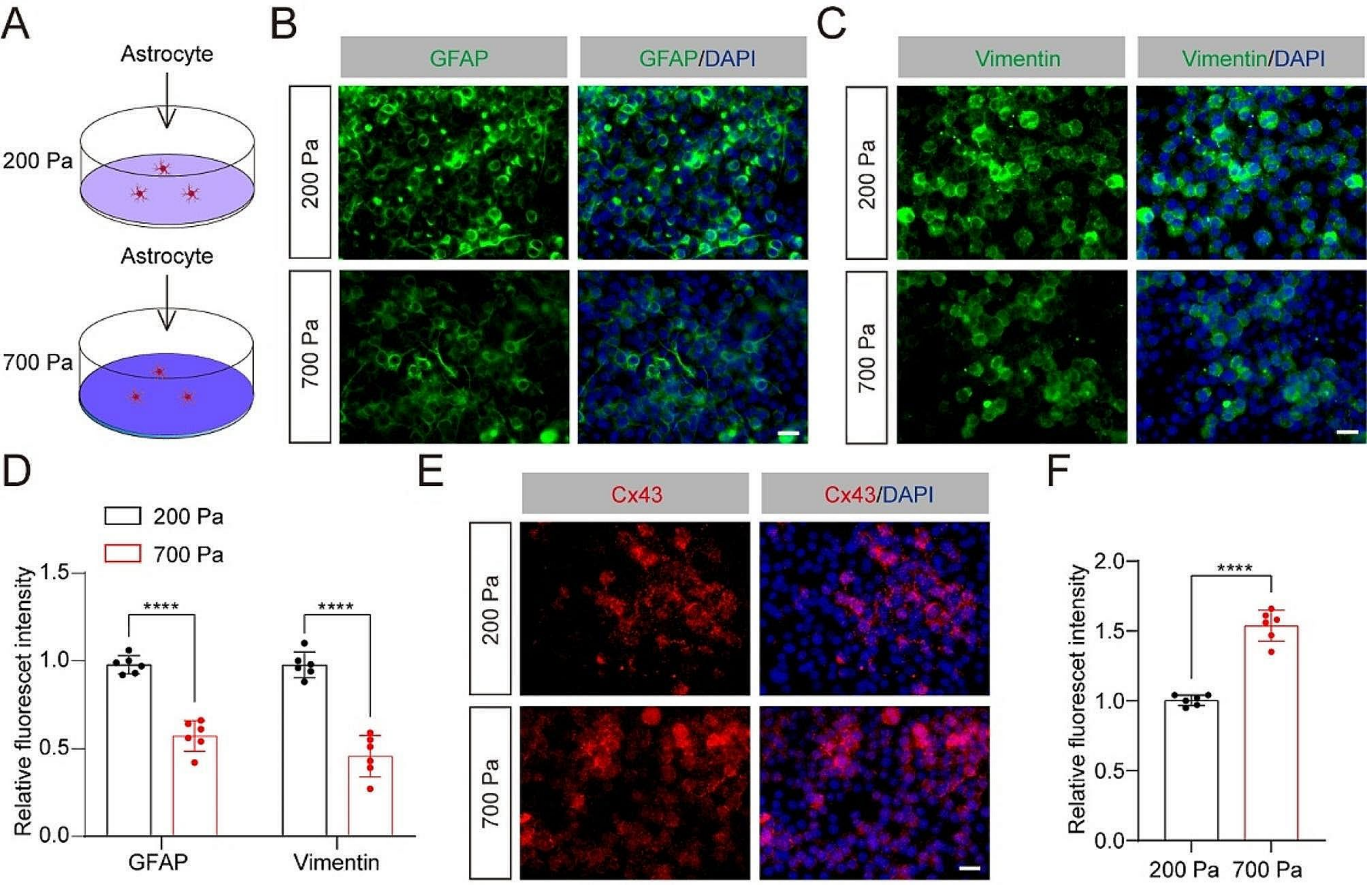
Physiologic changes in substrate stiffness alter Cx43 expression in astrocytes in vitro. (**A**) Experimental schematic diagram of astrocytes cultured on the substrate with 200 and 700 Pa. (**B**, **C**) Typical immunofluorescence images for the astrocyte marker GFAP (green, **B**) and vimentin (green, **C**) in different stiffness. Scale bar: 20 μm. (**D**) Quantitative analysis of the relative GFAP and vimentin intensity. (**E**) Typical immunofluorescence images for the Cx43 (red) in different stiffness. (**F**) Quantitative analysis of the relative Cx43 intensity. *n* = 6 independent cell cultures in (**D**) and (**F**). ****P < 0.0001 by Student’s *t* test

To more directly ascertain whether the spinal cord tissue stiffness affects the expression of Cx43 in astrocytes, we assessed the levels of Cx43 in astrocytes in vitro by changing substrate stiffness based on mechanical properties we measured from the spinal cord [25]. Astrocytes were cultured on polyacrylamide hydrogels with elastic moduli of 200 Pa to mimic the rigidity in the injured spinal cord of the control mice at 7 and 14 dpi. Additionally, hydrogels with elastic moduli of 700 Pa were utilized to simulate the rigidity in the injured spinal cord of the mice treated with PLX5622 or the *Fascin-1* CKO mice at 7 or 14 dpi (Fig. 6A). We conducted staining for GFAP and vimentin and found astrocytes expressed lower level of these two proteins in 700 Pa compared to 200 Pa (Fig. 6B-D). The data further verify that the level of GFAP and vimentin has a high correlation with tissue stiffness of the spinal cord. In addition, astrocytes in 700 Pa showed a higher level of Cx43 than in 200 Pa (Fig. 6E, F). Thus, these results directly confirm that enhanced tissue stiffness in vitro, corresponding to limited tissue softening after SCI, suppresses the level of GFAP and vimentin while upregulating Cx43.

### Fascin-1 deletion in microglia enhances myosin activity after SCI

**Fig. 7:**
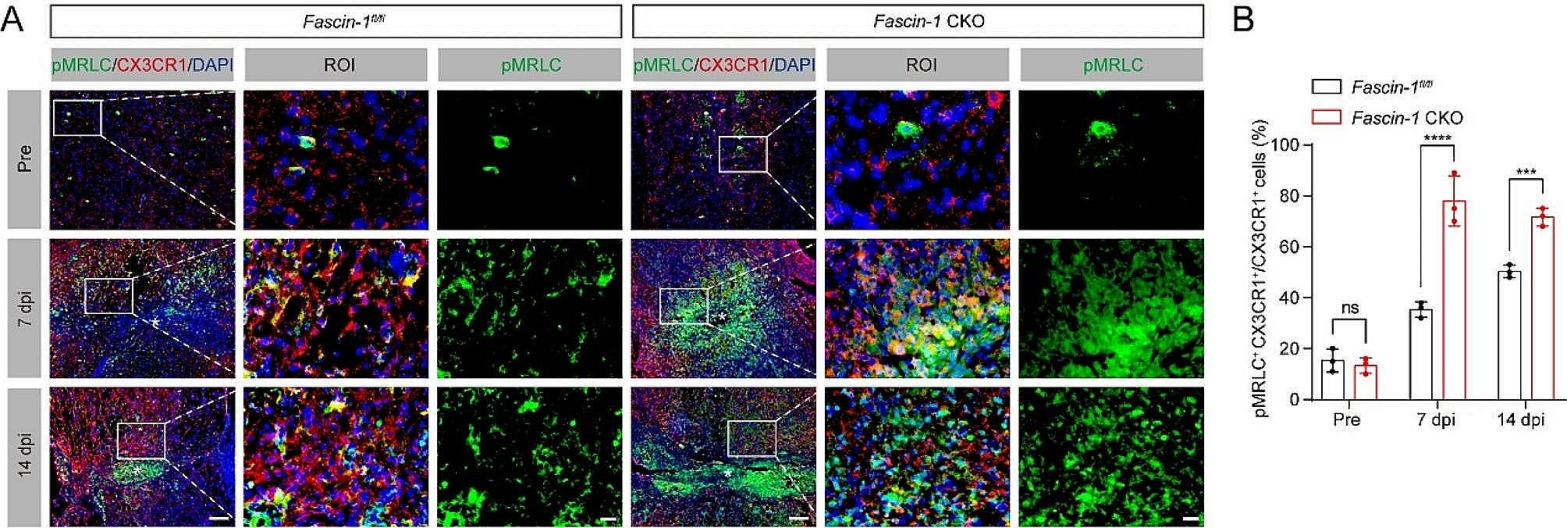
Fascin-1 deletion in microglia enhances myosin activity after SCI. (**A**) Sagittal immunofluorescence images of pMRLC (green), CXRCR1 (red), and DAPI (blue) from *Fascin-1*^*fl/fl*^ and *Fascin-1* CKO mice before SCI and at 7 and14 dpi. The regions of interest (ROI) represent the boxed regions on the left. The lesion core was marked with the asterisks. Scale bar: higher magnification, 20 μm; low magnification, 100 μm. (**B**) Percentage quantification of pMRLC^+^CX3CR1^+^ cells in relation to the overall CX3CR1^+^ cells. *n* = 3 animals in (**B**). ns, no significance; ***P < 0.001, ****P < 0.0001 by two-way ANOVA

Previous data demonstrated that Fascin-1 inhibits myosin activation to facilitate border cell migration and control substrate stiffness in *Drosophila* [19]. Phosphorylation on the regulatory light chain subunit of myosin (pMRLC) indicates its activation [38]. We speculated that Fascin-1 limits myosin activity, thus promoting migration of microglia and regulating tissue stiffness after SCI. To address our hypothesis, we first stained the tissues using an antibody against pMRLC. According to the immunofluorescence findings, the proportion of pMRLC^+^CX3CR1^+^ cells to the overall CX3CR1^+^ cells in *Fascin-1* CKO mice was higher than in *Fascin-1*^*fl/fl*^ mice at 7 and 14 dpi after SCI (Fig. 7A, B). These results suggest that Fascin-1 deletion in microglia upregulates myosin activity after SCI, and the Fascin-1/Myosin signaling in microglia may regulate tissue stiffness after SCI.

### Pharmacological inhibition myosin activity rescues microglial migration and mechanical changes in *Fascin-1* CKO mice

**Fig. 8:**
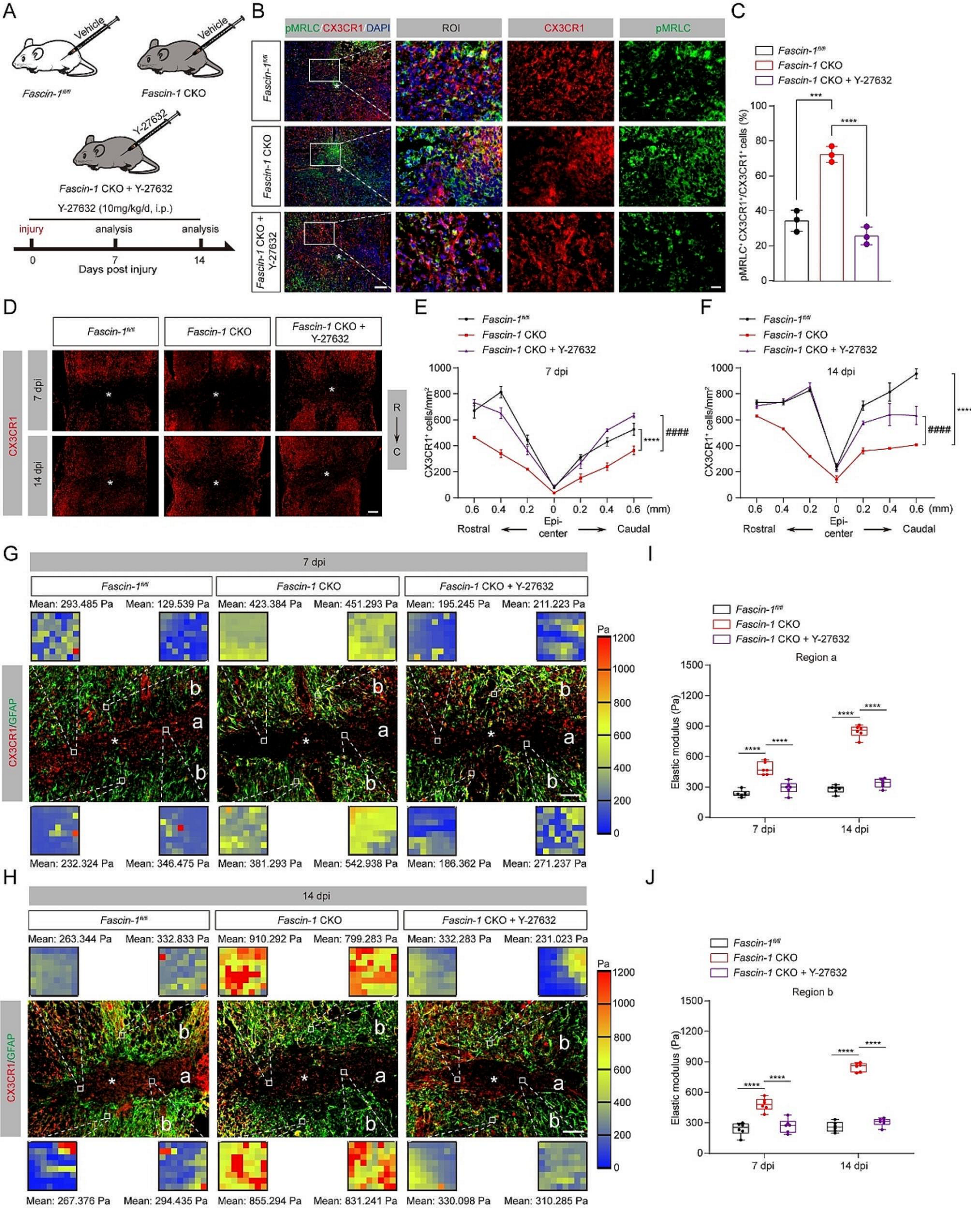
Reducing myosin activity rescues microglial migration and mechanical changes in *Fascin-1* CKO mice. (**A**) Schematic of the *Fascin-1*^*fl/fl*^ and *Fascin-1* CKO mice treatment with Y-27,632 or vehicle. The myosin activity inhibitor Y-27,632 was administered by intraperitoneal injection daily from 4 h after injury until 14 dpi. (**B**) Sagittal immunofluorescence images of pMRLC (green), CX3CR1(red), and DAPI (blue) from *Fascin-1*^*fl/fl*^ and *Fascin-1* CKO mice treated with vehicle and Y-27,632 at 7 dpi. Scale bar: higher magnification, 20 μm; low magnification, 100 μm. (**C**) Percentage quantification of pMRLC^+^CX3CR1^+^ cells in relation to the total number of CX3CR1^+^ cells. (**D**) Sagittal immunofluorescence images of CX3CR1 (red) in different groups as described above at 7 and 14 dpi. Scale bar: 20 μm. (**E, F**) Quantification of the density of microglia in different distance from the lesion epicenter at 7 (**E**) and 14 dpi (**F**). (**G**, **H**) Sagittal immunofluorescence images for microglia (CX3CR1, red) and astrocyte (GFAP, green) in different groups as described above at 7 (**G**) and 14 dpi (**H**). Region a represents the GFAP^−^ lesion core, and region b represents the GFAP^+^ penumbra region. Scale bar: 100 μm. (**I**, **J**) Comparison of the elastic properties of lesion core (a, **I**) and penumbra region (b, **J**) in different groups as described above in (**I**) and (**J**). The lesion core was marked with the asterisks. *n* = 3 animals in (**C**), (**E**), (**F**), (**I**), and (**J**). ***P < 0.001, ****P < 0.0001 by one-way ANOVA in (**C**). ****P < 0.0001 by two-way ANOVA in (**E**), (**F**), (**I**), and (**J**)

To investigate how Fascin-1 influences microglial migration and tissue stiffness by inhibiting myosin activation, we next used a pharmacological inhibitor of myosin activity (Y-27,632) to treat *Fascin-1* CKO mice as previously reported in other studies [19, 39], and assessed the influence on microglial migration (Fig. 8A). In vitro, the transwell and scratch assays showed that the migration capability of primary microglia was enhanced by the inhibition of myosin activity using Y-27,632 (Supplementary Fig. 2). Y-27,632 treatment abolished the increase of myosin activity induced by conditional deletion of Fascin-1 in microglia (Fig. 8B, C). Subsequently, we assessed microglial migration according to the number of CX3CR1^+^ microglia per mm^2^ at different distances from the lesion epicenter in three groups of mice [40]. The results revealed that Y-27,632 recused microglial migration in *Fascin-1* CKO mice (Fig. 8D-F). We then sought to determine the influence of inhibiting myosin activity on the changes in tissue stiffness induced by Fascin-1 deletion. We observed a significant tissue softening in *Fascin-1* CKO mice with Y-27,632 treatment at 7 and 14 dpi after SCI (Fig. 8G, H, right panel; Supplementary Fig. 1E, right panel). The elastic modulus of *Fascin-1* CKO mice with Y-27,632 treatment was lower than that of *Fascin-1* CKO mice with vehicle treatment (Fig. 8G-J, Supplementary Fig. 1E, F). These results demonstrate that pharmacological inhibition of myosin activity rescues microglial migration and mechanical changes in *Fascin-1* CKO mice after SCI.

### Reducing myosin activity rescues increased tissue pathology and poor functional recovery in *Fascin-1* CKO mice

**Fig. 9:**
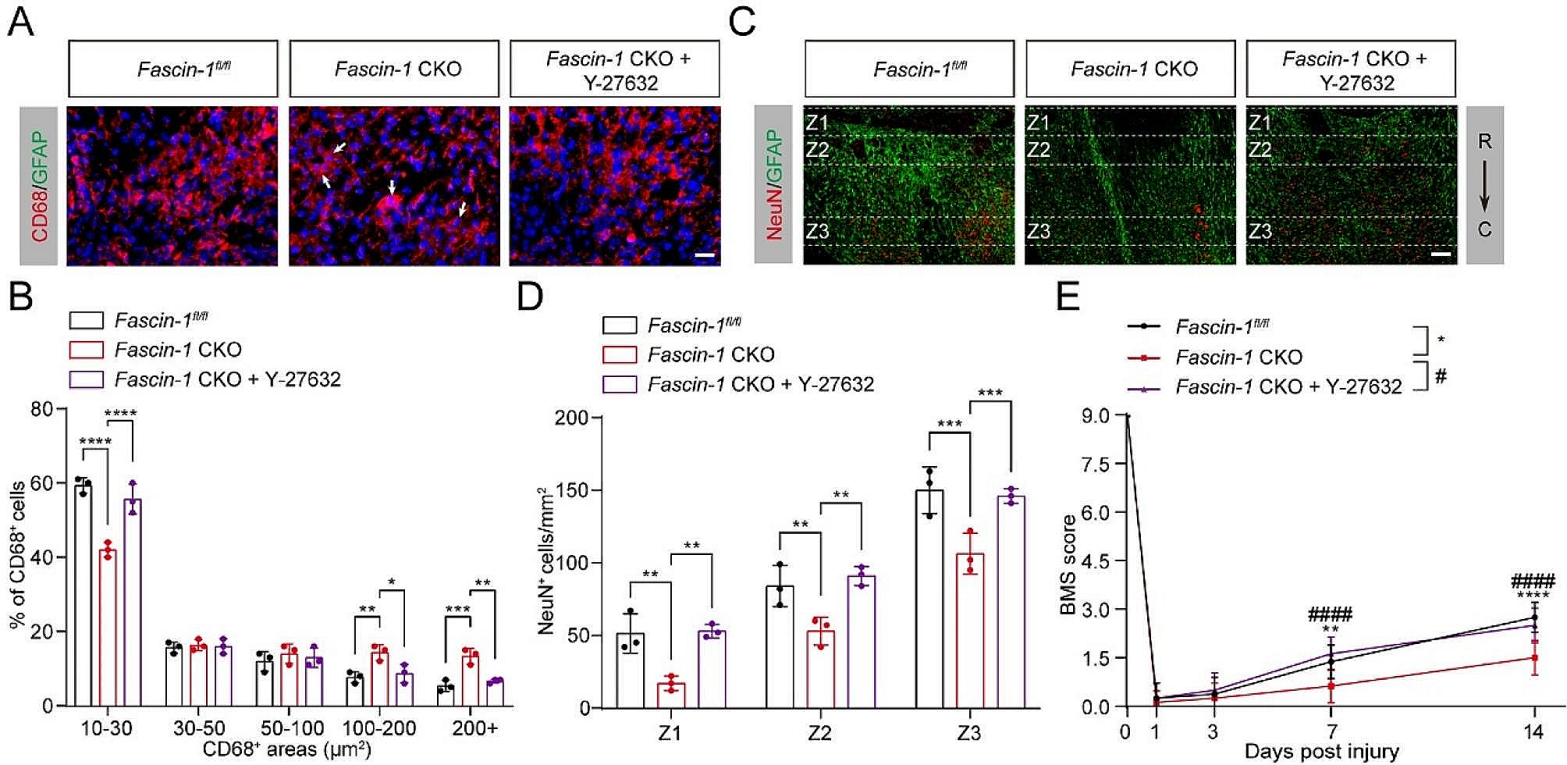
Reducing myosin activity rescues inflammation, neuron survival, and functional recovery in *Fascin-1* CKO mice. (**A**) Typical immunofluorescence images of CD68 (red) in the penumbra region in *Fascin-1*^*fl/fl*^ mice treated with vehicle and *Fascin-1* CKO mice treated with vehicle and Y-27,632 at 14 dpi. The nuclei were marked with DAPI (blue). Scale bar: 20 μm. (**B**) Reducing myosin activity rescues the size of CD68^+^ cells (red) in *Fascin-1* CKO mice. (**C**) Sagittal immunofluorescence images of NeuN^+^ neurons (red) and GFAP^+^ astrocyte (green) in Z1-Z3 zones neighboring the central lesion core in three groups at 14 dpi. Scale bar: 200 μm. (**D**) Quantitative analysis of NeuN^+^ neurons. (**E**) The BMS score was used to assess locomotor function at 0, 1, 3, 7, and 14 dpi in three groups.*n* = 3 animals in (**B**) and (**D**). *n* = 8 animals in (**E**). *P < 0.05, **P < 0.01, ***P < 0.001, ****P < 0.0001, ^####^*P* < 0.0001 by two-way ANOVA

We next assessed the consequences of limiting myosin activation by Y-27,632 treatment in *Fascin-1* CKO mice after SCI. Y-27,632 restores the increased size of the CD68^+^ phagolysosomes in *Fascin-1* CKO mice (Fig. 9A, B). The number of viable NeuN^+^ neurons in the Z1-Z3 zones of *Fascin-1* CKO mice with Y-27,632 treatment was significantly increased than the *Fascin-1* CKO mice with vehicle treatment (Fig. 9C, D), indicating limiting myosin activation rescues the decreased neuron survival in *Fascin-1* CKO mice. Accordingly, the BMS score showed that Y-27,632 treatment ameliorated the damaged locomotor function in *Fascin-1* CKO mice (Fig. 9E). Our results indicate that reducing myosin activity rescues increased tissue damage and ameliorates functional recovery caused by Fascin-1 deletion in microglia after SCI.

## Discussion

**Fig. 10:**
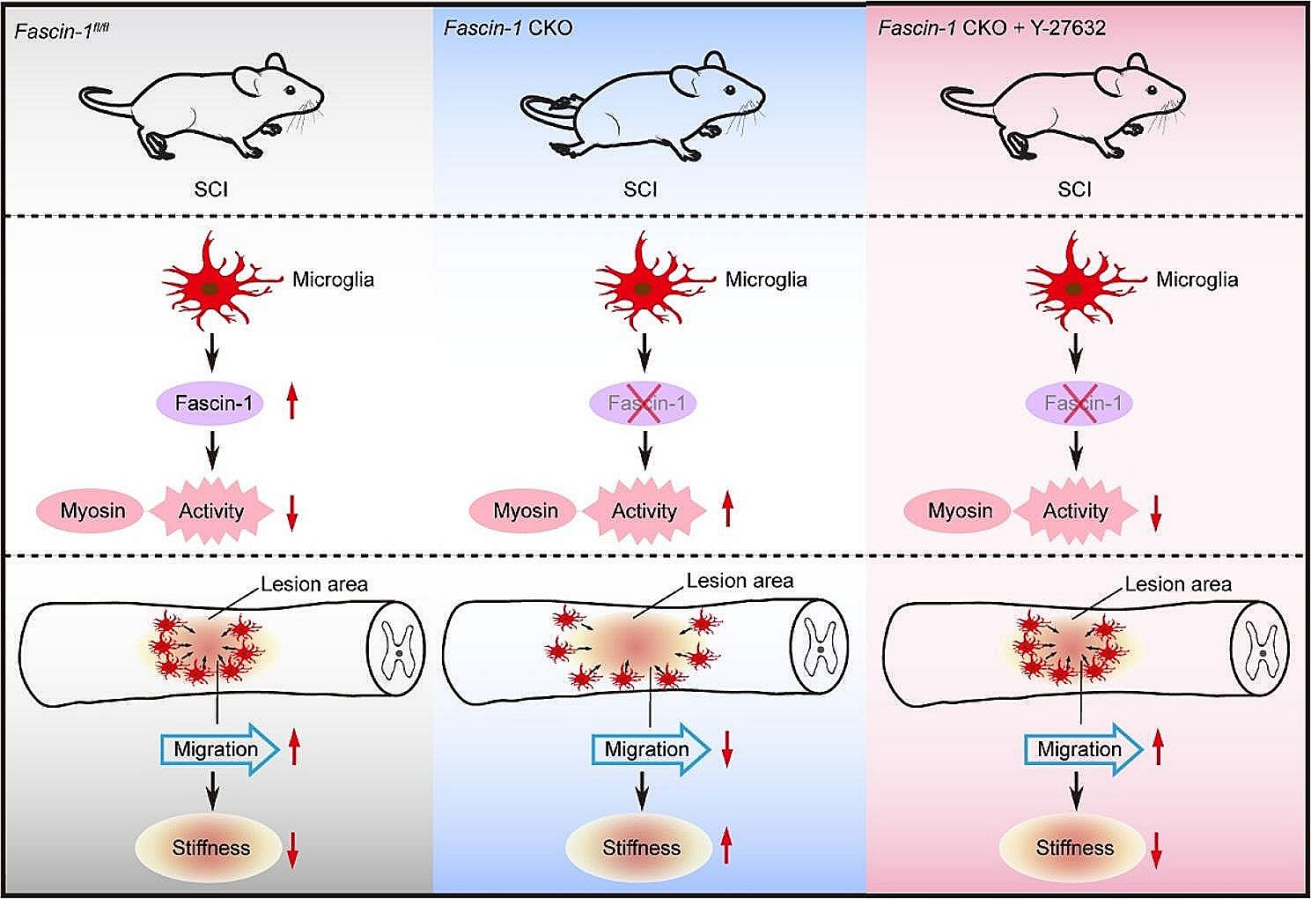
Schematic diagram for Fascin-1 limiting myosin activity to control spinal cord stiffness during microglial migration. In *Fascin-1*^*fl/fl*^ mice, Fascin-1 inhibits myosin activity to promote microglial migration. Microglia are involved in injury core mechanical signaling changes that soften the tissue. In *Fascin-1* CKO mice, myosin activity in microglia is increased, driving microglial migration down. The decrease of migration restrains spinal cord tissue softening. Reducing myosin activity rescues microglial migration and tissue softening in *Fascin-1* CKO mice

The interaction between immune cells and astrocytes mediates the formation of glial scar, promoting spinal cord softening at the injury site after SCI. This overlooks the role of microglia in spinal cord softening process. In this study, we found that Fascin-1 inhibits myosin activation to promote microglial migration and control mechanical characterization after SCI (Fig. 10). Microglia depletion or Fascin-1 deletion in microglia limits tissue softening and alters mechanical characterization, results in increased size of the CD68^+^ inflammatory cells, less neuron survival, and poor functional recovery. Moreover, pharmacological inhibition of myosin activity rescues the changes induced by Fascin-1 deletion in microglia.

Numerous studies reveal that migrating cells control substrate stiffness in their local environment. In the previously established stab injury model, the softening of glial scar correlated well with glial intermediate filaments containing GFAP and vimentin, and specific composition of extracellular matrix (ECM) in glial scar containing collagen and proteoglycans, whose expression is upregulated after injury [11]. In our study, a crush model consistent with clinical SCI was used and showed there was no obvious mechanical distinction between the GFAP^−^ lesion core and GFAP^+^ penumbra region after SCI, which can be partly explained by the distribution of microglia in both regions. Similarly, we found that the glial scar were destroyed by microglia depletion after SCI, suggesting that microglia play a crucial role in spinal cord softening process. Moreover, migrating cancer cells can transmit sustained traction to the microenvironment by myosin contractility and β1 integrin ligation [41], and the cells that migrate together exert greater force on their surroundings compared to individual cells, inducing fiber alignment in the ECM, ultimately altering substrate stiffness [42]. ECM spans the lesion core containing fibronectin and the collagen and penumbra region containing laminin after SCI [43]. Here, we found that Fascin-1 inhibits myosin activity to promote microglial migration and force transmission between the migrating microglia and the spinal cord tissues. The softening of spinal cord tissues is likely associated with microglial migration. However, the mechanism of microglial migration in controlling tissue stiffness remains unclear. Further exploring the ligand and receptor or other forms of signaling pathways between microglia and their environment will expand our understanding of how microglial migration regulates substrate mechanical characterization.

Scar tissue in the body tends to be more rigid than the neighboring healthy tissue [44, 45]. Here, we showed that the spinal cord does not experience stiffening but instead undergoes a softening process at 7 and 14 dpi. This acute drop in mechanical stiffness agrees with a previous report that brain tissue and spinal cord tissue stiffness decreased in a rat model 1–3 weeks after brain stab injury or spinal cord crush injury [11]. The softening of tissues is not only restricted to brain injury and SCI, but acute inflammation also decreases brain tissue stiffness in experimental autoimmune encephalomyelitis [46]. However, some other studies considered that SCI leads to chronic mechanical stiffening [47]. The chronic stiffening reflects fibrotic scar formation, which has yet to be fully established in acute and subacute lesions [48]. Moreover, we consider the discrepancies in mechanical stiffness of injured spinal cord tissues most likely due to the choice of experimental model. Indeed, different SCI models have advantages and limitations [49], and the particular criteria for various animal models could be further improved.

Fascin-1 is highly expressed and promotes invasion and metastasis in tumor cells [50, 51], and the dephosphorylation of fascin-1 contributes to adaptation to mechanical stress in podocytes [52]. Fascin-1 shows an increase in expression in microglia of spinal cord and dorsal root ganglions in a rat model of sciatic nerve injury [53]. Our previous research demonstrated that Fascin-1 regulates microglial migration in vitro [18, 54], which is confirmed in this study in vivo. Here, the *CX3CR1*^*cre*^ mouse line was used for microglia-specific Fascin-1 knockout, and the limitation is that this mouse line also targets CX3CR1-expressing perivascular and blood-derived macrophages, in addition to microglia [55]. Moreover, *CX3CR1*^*cre*^ mice have an extremely low probability of leakage into neurons [56]. Our previous study has confirmed the specifical expression of Fascin-1 in microglia after SCI [18], which partly supports the reliability of our model. Certainly, additional work is needed to establish an inducible *CX3CR1*^*cre*^ or *Hexb*^*cre*^ mouse line to achieve Fascin-1 ablation only in microglia [57]. In addition, Fascin-1 is an actin cross-linking protein and prevents myosin binding to actin, thereby, restricts myosin activity [58, 59]. F-actin bundling function is inhibited by phosphorylation of Fascin-1 at serine 52 [60]. The mechanism of Fascin-1 limits myosin activity is needed to fully elucidate in further experiments. The previous research has suggested that Fascin-1 limits myosin activity to facilitate migration and regulate substrate stiffness in *Drosophila* border cells [19]. According to our SCI model in mice, reducing myosin activity rescues inhibited migration of microglia in *Fascin-1* CKO mice, suggesting myosin participates in Fascin-1 mediated microglial migration to control mechanical characterization in mammalian SCI.

Harmful mechanical signals induce the damage of tissues, resulting in chronic inflammatory diseases such as osteoarthritis and lumbar degeneration [61, 62]. Mechanosensing is critical for neuron growth in CNS, such as redirecting axons clearly in vivo and in vitro under stiffness gradients [63]. Cx43 gap junctions and hemichannels are mechanoreceptors, such as inducing cardiomyocytes in response to mechanical stress [64, 65]. This Cx43 may regulate inflammation, neural protection, and functional recovery in CNS [32–34]. Our research showed that limited tissue softening by either microglia depletion or Fascin-1 deletion in microglia upregulates Cx43, and thereby increasing the size of CD68^+^ inflammatory cells, inhibiting neuron survival and impairing functional recovery after SCI. Thus, manipulating Cx43 in mechanotransduction may be an effective therapeutic approach for repairing the injured CNS.

Microglia play a crucial role in facilitating function recovery and protecting against neuronal injury at acute and sub-acute times after SCI [13]. There is ample evidence that microglia restore lesion homeostasis through coordinating cellular interactions in different SCI models. Our results showed that depleting microglia damaged the glial scar and altered the mechanical characterization of the injured spinal cord, ultimately impeding the process of functional recovery after SCI. Mechanistically, Fascin-1 limits myosin activity in microglia to provide mechanotransduction to the CNS microenvironment. However, constructing the specific model of changing tissue stiffness in the spinal cord to clarify the effect of mechanical signals on functional recovery after SCI requires further exploration. Activated and proliferating microglial cells play a protective/repairing role in the injured spinal cord within the first week post-injuryc [13]. The upcoming work will delve deeper into examining the role of Fascin-1 in regulating mechanical signal alterations during the chronic phase of spinal cord injury recovery. Overall, it has been rarely reported that microglia regulate the mechanical signals after SCI, this emerging role of microglia in controlling mechanical characterization should be manipulated in future research to repair the injured CNS.

## Conclusion

The present study reveals that Fascin-1 limits myosin activity in microglia to regulate the mechanical characterization of the injured spinal cord, which alleviates tissue pathology and promotes locomotor function recovery after SCI. Targeting Fascin-1/Myosin signaling in microglia to control mechanical characterization should be considered in future research to facilitate recovery of CNS diseases ultimately.

### Electronic supplementary material

Below is the link to the electronic supplementary material.

Supplementary Material 1

## Data Availability

No datasets were generated or analysed during the current study.

## Abbreviations

CNS: Central nervous system
SCI: Spinal cord injury
ECM: Extracellular matrix
GFAP: Glial fibrillary acidic protein
CSF1R: Colony stimulating factor 1 receptor
AFM: Atomic force microscopy
DMSO: Dimethylsulfoxide
Dpi: Days post-injury
PBS: Phosphate buffered saline
DSA: Dovine serum albumin
Cx43: Connexin 43
pMRLC: Myosin regulatory light chain
DAPI: 4′,6-Diamidino-2-phenylindole, dilactate
PFA: Paraformaldehyde
FBS: Fetal bovine serum
BMS: Basso mouse scale
MDMs: Monocyte-derived macrophages
