# Supplementary material for: Fascin-1 limits myosin activity in microglia to control mechanical characterization of the injured spinal cord
Source: J Neuroinflammation. 2024 Apr 10;21:88. doi: 10.1186/s12974-024-03089-5 (PMC11005239; doi:10.1186/s12974-024-03089-5)
Supplement: Supplementary file 1 — Supplementary Material 1 [file 12974_2024_3089_MOESM1_ESM.docx]

**
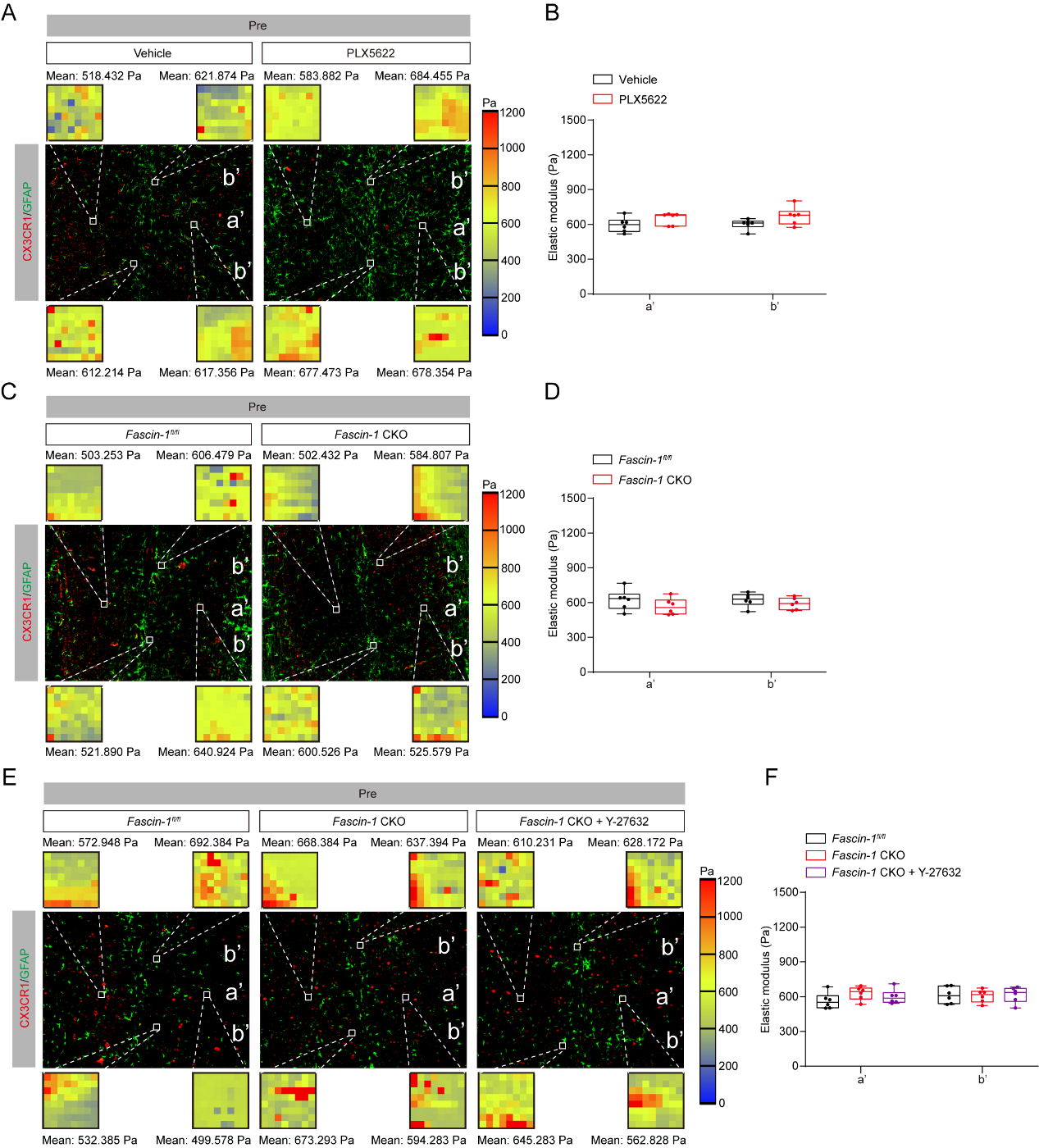
**

**Supplementary Fig. 1** There is no notable mechanical distinction of the elastic properties in different groups of uninjured mice. **A**, **C**, **E** Sagittal immunofluorescence images for microglia (CX3CR1, red) and astrocyte (GFAP, green) in different groups of uninjured mice. Scale bar: 100 μm. The positions of a’ and b’ exhibit similarities with the regions of the damaged tissues in relation to the spinal cord midline. **B**, **D**, **F** Comparison of the elastic properties of regions a’ and b’ in different groups of uninjured mice in (**A**), (**C**) and (**E**). n = 3 animals in (**B**), (**D**), and (**F**).


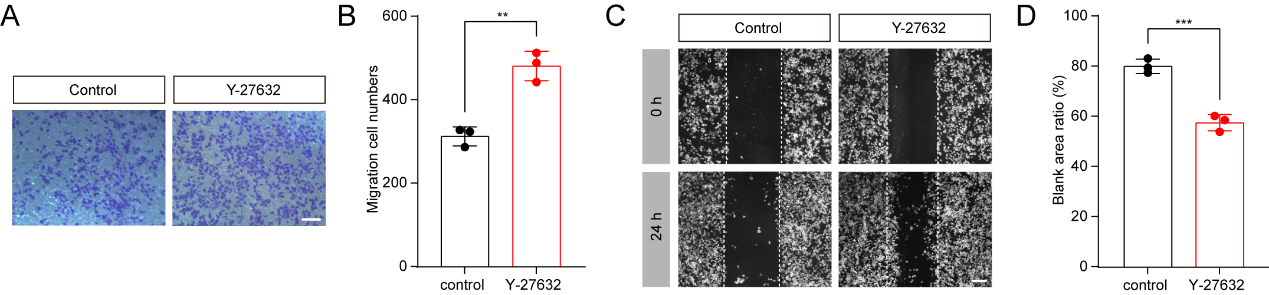


**Supplementary Fig. 2** Inhibiting myosin activity promotes microglial migration in vitro. **A**, **C** Primary microglia were treated with control or Y-27632 for 24 hours. Cell migration was recorded after an additional 24 hours in the transwell assays (**A**) and the scratch assay (**C**). Scale bar: 20 μm. **B** Analyzing the quantity of transmembrane cells. **D** Quantification of the blank area ratio in the scratch assay. n = 3 independent cell cultures in (**B**) and (**D**). ***P*<0.01, ***P<0.001 by Student’s t test.
